# Special Issue on “Cellular and Molecular Mechanisms Underlying the Pathogenesis of Hepatic Fibrosis”

**DOI:** 10.3390/cells9051105

**Published:** 2020-04-29

**Authors:** Ralf Weiskirchen

**Affiliations:** Institute of Molecular Pathobiochemistry, Experimental Gene Therapy and Clinical Chemistry (IFMPEGKC), RWTH University Hospital Aachen, D-52074 Aachen, Germany; rweiskirchen@ukaachen.de; Tel.: +49-(0)241-8088683

## Abstract

This Special issue contains 48 contributions highlighting novel findings and current concepts in basic and clinical liver fibrosis research. These articles emphasize issues on pathogenesis, cellular mediators, modulators, molecular pathways, disease-specific therapies, scoring systems, as well as novel preclinical animal models for the study of liver fibrogenesis. This editorial aims to briefly summarize the content of these papers.

## 1. Introduction

Liver fibrosis and associated complications bring substantial medical and economic burdens worldwide [1]. This process is characterized by excessive accumulation of extracellular matrix (ECM) expressed by activated hepatic stellate cells (HSC) and driven by a large variety of mediators that become released or expressed during hepatic injury. During recent decades major advances in the knowledge of molecular and cellular mechanisms relevant in hepatic fibrogenesis were achieved. However, pharmacological intervention with anti-fibrotic acting drugs is complicated and often effective only in preclinical animal models [2]. This is due to the fact that the pathogenic sequence during fibrogenesis is complex involving several cell types and a broad spectrum of soluble factors including chemokines, cytokines, hormones, and a variety of non-peptide mediators [3]. Liver cirrhosis is the end-stage of liver fibrosis and represents a major risk factor for the development of hepatocellular carcinoma (HCC).

Worldwide, liver research aims to increase basic knowledge, improve diagnostics, and try to translate experimental findings into new treatment modalities. However, based on the overall complexity of molecular and cellular events changing during initiation and progression of hepatic fibrosis, research of scientists and healthcare professionals covers a wide array of different and complex issues. Nevertheless, there is great hope that basic and translational research in this area will lead to new therapies in the near future.

**Figure 1:**
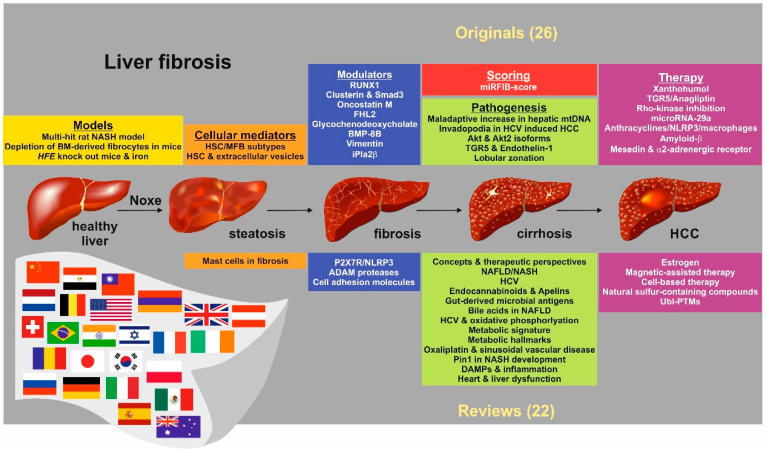
Topics covered in this Special Issue. The Special Issue “*Cellular and Molecular Mechanisms Underlying the Pathogenesis of Hepatic Fibrosis*” includes 26 original and 22 review articles written by 319 authors from 25 countries. These contributions discuss aspects of basic, clinical and translational research in the field of liver fibrosis. Special emphasis is given on the themes listed. Abbreviations used are: ADAM, a disintegrin and metalloproteinase; Akt1/2, serine/threonine-protein kinase 1/2; BM, bone marrow; BMP-8B, bone morphogenetic protein-8B; DAMPs; damage-associated molecular patterns; FHL2, four-and-a-half LIM-domain protein 2; HCC, hepatocellular carcinoma; HCV, hepatitis C virus; *HFE*, gene encoding the homeostatic iron regulator; HSC, hepatic stellate cell(s); iPla2β, Phospholipase A2; MFB, myofibroblast(s); mtDNA, mitochondrial DNA; NAFLD, nonalcoholic fatty liver disease; NASH, nonalcoholic steatophepatitis; NLRP3, NLR family pyrin domain-containing 3; Pin1, peptidyl-prolyl cis/trans isomerase, NIMA-interacting 1; P2X7R, purinergic receptor P2X; RUNX1, Runt-related transcription factor 1; TGR5, Takeda G-protein-coupled receptor 5; Ubl-PTMs, Ubiquitin-like post-translational modifications.

In this Special Issue, leading experts from the field share new findings and concepts of the current state in experimental and clinical liver fibrosis research. In total 319 scientists from 25 countries (Armenia, Australia, Austria, Belgium, Brazil, China, Egypt, France, Germany, Great Britain, India, Japan, Korea, Mexico, Poland, Russia, Spain, Switzerland, Ireland, Israel, Italy, Romania Taiwan, The Netherlands, USA) contributed original or review articles about current perspectives and findings in hepatic fibrosis (Figure 1). These articles cover a wide range of aspects showing that this fascinating field has made conceptual advances that hopefully will lead to novel treatment options in the near future.

## 2. General Aspects of Fibrogenesis

General mechanistic concepts of liver fibrosis and therapeutic consequences and perspectives resulting thereof are summarized by Roehlen and colleagues [4]. They introduce the major cell types (hepatocytes, HSC, liver macrophages, lymphocytes), factors (e.g., gut dysbiosis; reactive oxygen species (ROS); inflammasome) and signaling pathways (platelet-derived growth factor, PDGF; transforming growth factor-β, TGF-β; Wnt/catenin-β) that orchestrate hepatic fibrosis. Moreover, they provide information about major causes and triggers relevant for the initiation of fibrosis and therapeutic strategies [4]. Reading this review is a solid starting point into this Special Issue, providing a large list of key references relevant for this topic. One more specialized review summarizes the current knowledge in the pathogenesis of nonalcoholic fatty liver disease (NAFLD) and nonalcoholic steatohepatitis (NASH) [5]. The authors provide a comprehensive overview about risk factors, symptoms, diagnosis, and treatment options of nonalcoholic liver disease. Moreover, blood biomarkers, multiparametric non-invasive fibrosis test panels, and imaging strategies presently used in clinical practice to assess fibrosis are compared and discussed. Another review discusses the molecular mechanisms responsible for the progression of liver fibrosis in chronic hepatitis C virus (HCV)-infected patients [6]. The authors discuss how HCV infections modulate microRNAs and long non-coding RNAs expression patterns in HCV-infected hepatocytes and their potential to affect profibrogenic HSCs. Other aspects of HCV infection are summarized by Gerresheim and colleagues [7]. In their contribution, they discuss that the metabolic switch and early programming of infected cells to cancer cells can be regarded as a mechanism by which the virus receives advantages for its ongoing replication. The authors developed a novel interesting hypothesis in which the anabolic metabolites resulting from enhanced conversion of glucose to lactate during aerobic glycolysis (“Warburg effect”) is a collateral event rather than a requirement for cancer cell growth. Thematically related is an article compiled by [8]. The authors used both sophisticated functional and genomic screens to systematically elucidate the molecular mechanisms contributing to the HCV-invasiveness of HCC cells. A model is suggested by which HCV controls a unique expression signature resulting in invadopodia precursor formation and stimulation of epidermal growth factor receptor signaling, thereby modulating degradation of extracellular matrix compounds, invasiveness, and metastasis. Another contribution highlights factors modulating hepatic fibrosis. In one contribution, the multi-systemic nature of the gut-liver axis in health and disease is summarized [9]. The authors discuss the impact of microbial translocation for chronic liver disease, cell-specific responses of the liver to intestinal antigens, and mechanistic insights into how these antigens drive the development and progression of hepatic inflammation and fibrosis.

## 3. Bile Acids in Hepatic Fibrosis

Five contributions address aspects of bile acid functions in hepatic health and disease. There is ample evidence that alterations in bile acid metabolism are closely linked to NAFLD and NASH. The review by Gottlieb and Canbay focusses on the complexity of the liver-bile-intestinal axis, promising clinical targets to tackle bile acid dysbalances in NAFLD/NASH, and pharmaceutical strategies evolving positive effects on bile acid composition [10]. Klindt and coworkers comparatively fed wild type and mice disrupted for the G-protein-coupled bile acid receptor (Gpbar1 or TGR5) with a diet containing 1% lithocholic acid for 84 hours [11]. The authors found that the supplementation provoked an enrichment of pathways associated with inflammation and matrix remodeling. Interestingly, compared to wild type mice, animals lacking TGR5 further showed aggravated liver damage and elevated hepatic expression of endothelin-1, while activation of TGR5 reduced ET-1 expression in HSC and sinusoidal endothelial cells. These findings show the complexity of TGR5 signaling in the pathogenesis of liver fibrosis. The complexity of this pathway is further documented by Kaya et al. demonstrating that the TGR5 agonist oleanolic acid significantly improved glycemic status and attenuated intrahepatic steatosis in male diabetic rats that received intraperitoneal injections of porcine serum to induce liver fibrosis [12]. Interestingly, the authors found that the TGR5 agonist oleanolic acid inhibited the increase in the Firmicutes/Bacteroidetes ratio that is an indicator of dysbiosis related to metabolic syndromes. Hohenester and colleagues reported that the supplementation of the hydrophobic bile acid glycochenodeoxycholate in a murine model of inducible hepatocellular cholestasis (i.e., cholate-fed *Atp8b1*^G308V/G308V^) promoted liver fibrosis [13]. Since this primary bile salt accumulates intra-hepatically during cholestasis, the data confirm the important relevance of bile acid in the formation and modulation of hepatic insult. Mice lacking the calcium-independent phospholipase A2 (*iPla2β*) show exaggerated liver fibrosis when fed a methionine-choline-deficient diet. Ming and colleagues have now demonstrated that respective mice show severely disturbed endoplasmic reticulum phospholipid composition, increased unfolded protein response, suppression of farnesoid X-activated receptor (*Fxr*) expression, and exacerbated bile acids in the liver and blood combined with ductular proliferation [14]. Therefore, this study adds iPla2β to the list of factors that is relevant in bile acid homeostasis.

## 4. Therapy of Fat-Induced Hepatic Fibrosis

Currently, there are no efficient strategies for the treatment of NAFLD/NASH. However, there are some potential targets and early drug candidates in the pipeline of researchers. Inoue and co-workers present a comprehensive review of the function of *Pin1* in the pathogenesis of NAFLD/NASH [15]. This peptidyl-prolyl cis/trans isomerase enhances lipogenesis through an insulin-signaling dependent mechanism, suppression of thermogenic genes, and enhanced adipocyte differentiation induced by increased peroxisome proliferation-activated receptor γ (PPAR-γ) activity. The authors discuss how these features trigger superoxide production, ROS formation, and hepatic inflammation. As the authors mentioned, a selective *Pin1* inhibitor counteracting these activities would be of fundamental therapeutic use in the treatment of NAFLD/NASH. Mahli et al. showed that micellar solubilized Xanthohumol is more effective in improving metabolic syndrome and NAFLD symptoms than native Xanthohumol in a mouse model fed with a Western diet for 3 weeks [16]. As discussed, Xanthohumol representing a prenylated chalcone from female hop cones is able to scavenge ROS, interfere with Prostaglandin-endoperoxide synthase 2 (PTGS2 or COX-2) activity, and further modulate enzymes involved in carcinogen metabolism and detoxification. Therefore, the oral application of micellar solubilized Xanthohumol appears as a promising strategy to inhibit NAFLD progression.

## 5. Mediators and Pathways in Hepatic Fibrosis

Nowadays, the knowledge on molecular mechanisms by which individual genes modulate hepatic steatosis and fibrosis is growing steadily. Wang et al. report new findings about the role of vimentin in regulating HSC proliferation and migration during hepatic fibrogenesis in dimethylnitrosamine-exposed rat livers [17]. The authors present data showing that this intermediate filament protein not only provides a major architecture for maintaining the stability of cytoskeletal proteins, but also is crucial in the activation of the extracellular-signal regulated kinases ERK and AKT, which control proliferation, differentiation, and movement of HSC during hepatic fibrogenesis [17].

Another critical mediator involved in control of HSC migration is Oncostatin M (OSM). This is documented by experiments performed by Foglia and colleagues [18]. They found that OSM and its receptor (OSMR) are overexpressed in three murine models of NAFLD/NASH and in human patients suffering from NASH. In primary culture of human HSC/MFBs and in the immortalized human HSC cell line LX-2, OSM triggers cell migration by the activation of several fibrosis-related pathways. In a large number of different experiments the authors demonstrate that intracellular ROS formation, HIF-1α production, as well as increased expression of vascular endothelial growth factor-A (VEGF-A) are most likely the key drivers of OSM-induced migration in HSC/MFB [18].

Inflammation is one important driver and promoter of liver fibrosis. As outlined by Ignat et al., there are many different pathways and cells stimulating the release of pro-inflammatory cytokines during hepatic fibrogenesis [19]. In their contribution, the mechanisms and significance of inflammasome assembly in mediating sterile inflammation and are explained. It becomes clear and comprehensible how different inflammasome complexes can act as machineries creating self-perpetuating loops protecting against danger signals, oxidative stress and pathogen infection. However, if the particles become over-activated, it results in wound healing and fibrosis. Therefore, understanding of assembly, functionality and downstream signaling molecules of these multi-competent particles can open new opportunities in the treatment of inflammatory liver disease. In this context, regulatory aspects of the NLR family pyrin domain-containing 3 (NLRP3) inflammasome are discussed by Rossato and colleagues [20]. In their review article, the authors discuss that the activation of this inflammasome is driven by extracellular ATP resulting in the activation of a specific purinergic, ligand-gated ion channel termed P2X7. It becomes clear that the purinergic signaling pathway is highly complex and directly associated with inflammation in many liver cells. Damaged cells releasing ATP provoke activation of P2X7 and induce the NLRP3 inflammasome. As the authors highlight, these processes critically contribute to the induction of NAFLD and NASH [20].

During alcoholic liver disease, inflammation, cell proliferation, migration and fibrogenesis are further triggered by Akt serine/threonine kinase isoforms [21]. The authors conducted a large set of in vitro and in vivo studies in Kupffer cells, HSC, hepatocytes, and in an ethanol- and lipopolysaccharide-induced two-hit mouse model. While both Akt1 and Akt2 regulated fibrogenesis and proliferation in hepatocytes and HSC, only Akt2 differently regulated inflammation and migration. In contrast, Akt3 had no effects on these processes, suggesting that the participation of the different isoforms in the pathogenesis of hepatic disease, although sharing a high degree of structural homology, is markedly different [21].

The impact of oxaliplatin on the pathogenesis of porto-sinusoidal vascular disease (PSVD) is discussed by Puente et al. This cancer drug is commonly used for treatment of colorectal cancer. Like other platinum compounds, it evolves non-targeted cytotoxic defects that might rarely result in PSVD. In their review, the authors discuss the three underlying mechanisms (i.e., porosity of sinusoidal endothelium, hypoxia-induced hyperplasia, and obliteration of blood capillaries) through which oxaliplatin causes sinusoidal damage and increase portal pressure during PSVD [22].

Schmidt-Arras and Rose-John discuss fibrosis-associated pathways regulated by members of the ‘A Disintegrin And Metalloprotease’ (ADAM) family [23]. These are transmembrane multidomain proteolytic active proteins, targeting the ectodomains of type I and type II transmembrane proteins. In their review, the authors provide an excellent summary about pro- and anti-fibrogenic activities of ADAM proteases and therapeutic perspectives resulting thereof. Based on the versatile functions of this class of enzymes, the authors concluded that ADAMs offer a rich but unexplored source of opportunities for the treatment of hepatic fibrosis.

Hintermann and Christen review the literature on selectins, cadherins, integrins and members of the immunoglobulin superfamily of adhesion molecules in the context of hepatic fibrosis [24]. Particularly, their contribution highlights that these molecules are important for attracting liver-resident cells or patrolling leukocytes to the site of hepatic damage. As such these proteins have fundamental roles in matrix remodeling and angiogenesis. They modulate multifaceted interactions between cells, thereby fine-tuning the interplay of cells and extracellular matrix compounds in the fibrotic environment.

Similarly, novel important functions were shown for the four-and-a-half LIM-domain protein 2 (FHL2) supposed to act as a scaffolding protein. Sommer and colleagues now provide novel insights in the role of FHL2 in cholestatic liver injury with a special focus on hepatocellular damage and fibrosis [25]. The authors found that *Fhl2*-deficient mice develop significantly more hepatic damage and inflammation when subjected to bile-duct ligation than normal control mice. In HepG2 cells, the depletion of *Fhl2* affected expression of key enzymes of bile acid metabolisms and hepatocellular injury. These finding suggest that FHL2 is important for liver homeostasis and more than just a simple scaffolding protein.

In another contribution, Seo et al. found that clusterin reduces hepatic fibrosis. They could demonstrate that this multifunctional glycoprotein inhibits activation of HSC in a thioacetamide-induced liver fibrosis model and TGF-β signaling pathway and collagen expression in primary mouse HSC or immortalized human HSC cell line LX-2 [26]. Many previous studies have already shown that the expression of clusterin is increased in various disease conditions acting in the clearance of cellular debris. However, this study showing that clusterin mediates an inhibitory effect on HSC proofs another specific, antifibrotic activity of this molecular chaperone.

In hepatic fibrosis, the endocannabinoid and apelin systems are two of the multiple cell signaling pathways involved in the transformation of quiescent HSC into MFB. Both systems are upregulated during liver diseases and contribute to the development of hepatic fibrosis. The review of Melgar-Lesmes and colleagues summarizes the recent advances in understanding pathophysiologically relevant roles and their potential clinical relevance of both systems. Interestingly, the endocannabinoid and apelin pathways have marked dissimilarities and collaborate with each other in hepatic fibrogenesis [27].

## 6. Integration of ‘Omics’ and ‘Multit-Omics’ in Liver Fibrosis Research

Actually, a large number of studies use ‘omics’ or ‘multi-omics’ approaches to depict the complex sequence of events during hepatic fibrosis. The recent findings of metabolic alterations relevant to hepatic fibrosis obtained in selected studies were summarized by Chang and Yang [28]. The authors provide comprehensive information about carbohydrate-, amino acid-, and lipid-associated changes during the initiation and progression of fibrotic liver disease obtained in experimental and clinical studies. It is strengthened that the dynamic evolution of system biology has uncovered fibrotic signatures including alterations in energy generation, amino acid metabolism, retinoic acid homeostasis, serum lipid content, and mitochondrial respiration [28]. As Khomich et al. unanimously discuss, significant metabolic alterations in HSC are important for the transdifferentiation process [29]. They affect retinol and lipid metabolism, central carbon and nitrogen metabolism, redox status, and aspects of endoplasmic reticulum stress formation. It is obvious, that a more detailed understanding of these metabolic alterations is important for the development of strategies for pharmaceutical interventions in the sequence of events happening in these profibrogenic cells during pathogenesis of liver fibrosis [29].

## 7. Potential Drug Targets

In the Special Issue, several novel antifibrotic-acting drugs are discussed. Zhou and colleagues report that the selective RhoA/Rho-kinase (ROCK) inhibitor fasudil reduced the granuloma size, effectively promoted HSC apoptosis and inhibited expression of hepatic fibrogenic genes in mice infected with *Schistosoma japonicum* [30]. Importantly, the authors found that this vasodilator suppressed the activation and induced the apoptosis of CD4^+^ T cells, which mediate important immunopathogenic roles in Schistosomiasis.

Köse-Vogel et al. report novel findings about tissue-resident macrophages in promoting development of fibrosis in sterile tissue injury [31]. In their study, the anti-inflammatory properties of epirubicin on NLRP3- and Toll-like receptor 4 (TLR4)-mediated inflammation in phorbol 12-myristate 13-acetate-primed primary human peritoneal macrophages were investigated. This anthracycline drug acts by intercalating DNA strands, thereby triggering DNA cleavage by topoisiomerase II resulting in cellular apoptosis. The presented findings extend the spectrum of activity of this cancer drug showing anti-inflammatory capacity in reducing interleukin-1β (IL-1β) and tumor necrosis factor-α (TNF-α) release from macrophages when applied in low doses [31]. It will be interesting now to test if this drug can target inflammatory responses of tissue-resident macrophages in the context of inflammatory and fibrotic liver disease.

Milito et al. provide a reference collection on experimentally tested natural sulfur-containing antifibrotic compounds [32]. Most of them are pleiotropic molecules acting as scavengers for ROS and nitric oxide, or alternatively interfering with the activity of redox-sensitive pathways involved in the progression of hepatic fibrosis. Although the efficacy and safety of these experimental drugs is largely unknown, their beneficial effects in experimental models make their forthcoming translation from bench to bedside realistic [2,32].

## 8. MicroRNAs in the Pathogenesis of Hepatic Fibrosis

Two contributions in this Special Issue deal with aspects of microRNAs (miRNA) in liver fibrosis. These are non-coding, approximately 22 nucleotides long, single stranded RNA structures acting as posttranscriptional gene regulators. Lambrecht and coworkers performed miRNA-profiling in extracellular vesicles obtained from the conditioned medium of in vitro activated primary murine HSC. They produce a list of four miRNAs (miRNA-451a, miRNA-142-5p, Let-7f-5p, and miRNA378a-3p) that were significantly upregulated during HSC activation and in murine livers treated with carbon tetrachloride. Importantly, these molecules were also upregulated in serum obtained from patients with chronic alcohol abuse, chronic viral hepatitis infection, and NAFLD [33]. Based on these findings, the authors proposed a new fibrosis score (i.e., the miRFIB-score), in which these four miRNAs are combined with two already well-established fibrosis-associated miRNAs (miRNA-122-5p, miRNA-29a-3p) [33]. In another study, Lin and colleagues subjected wild type and miRNA-29a transgenic mice to a high fat diet to induce obesity, NAFLD, and fibrosis [34]. Interestingly, animals carrying the transgene showed reduced inflammation, weight gain, fat accumulation and liver weight. This was accompanied by reduced hepatic mitochondria and alterations in the expression profile of proteins facilitating the uptake of long-chain fatty acids. Consequently, the authors suggested this miRNA as a new potential candidate for a targeted therapy for treating fat-associated liver diseases such as NASH.

## 9. Posttranslational Protein Modification in Liver Fibrogenesis

Similar to miRNAs, posttranslational modification (PTMs) of proteins, and more specifically NEDDylation and SUMOylation ubiquitin-like (Ubls) modification have been shown to contribute to the progression of hepatic fibrosis [35]. Although the mechanisms underlying Ubl-PTM and the consequences of profibrogenic signaling are not well understood, increasing evidence show that Ubl-PTM are increased under stress conditions in a large variety of liver cells and in the setting of experimental and human liver fibrosis. Therefore, inhibition of Ubl-PTM might offer new therapeutic avenues to block or reverse hepatic fibrosis [35].

## 10. New Insights in Non-Alcoholic Fatty Liver Disease and Non-Alcoholic Steatohepatitis

The finding that men and postmenopausal women have a higher risk of NAFLD/NASH than premenopausal women is not well explained. The review by Lee and colleagues summarizes recent studies in which estradiol was supposed as the critical compound for developing these gender- and reproductive state-dependent differences in NAFLD/NASH [36]. They discuss estradiol as a hepatoprotective agent lowering hepatic fat accumulation, inflammation, and fibrosis. Kaur et al., investigated aspects of Runt-related transcription factor 1 (RUNX1) in the pathogenesis of NASH [37]. Commonly, RUNX1 is known as a transcription factor regulating the differentiation of hematopoietic stem cells into mature blood cells and angiogenesis. Interestingly, the authors found in a microarray study, a significant correlation between RUNX1 expression, several angiogenic genes, and severity of human NAFLD. Based on the fact that RUNX1 was further able to increase angiogenic activity in other endothelial cell systems, the authors speculate that RUNX1 might contribute to the pathogenesis of fatty liver diseases by modulating the angiogenic properties of endothelial cells resulting in aberrant liver angiogenesis and inflammation [37].

Mahli and colleagues investigated aspects of the bone morphogenetic protein-8B (BMP-8B) in NAFLD. This cytokine belongs to the TGF-β superfamily and is expressed in brown adipose tissues and the hypothalamus modulating thermogenesis and susceptibility to diet-induced obesity. Mahli et al. now show that BMP-8B expression is induced in hepatocytes when cultured in the presence of fatty acids, in a murine NAFLD model, and in NAFLD patients [38]. Moreover, the incubation with recombinant BMP-8B stimulated lipid accumulation, NF-κB signaling, pro-inflammatory gene expression, and uncoupled mitochondrial β-oxidation in hepatocytes, while siRNA-mediated blockade of BMP-8B expression ameliorated fatty acid-induced effects. These data demonstrate that BMP-8B acts in control of fat metabolism and storage by communicating with brown adipose tissue, brain, and of course the liver [38].

Malik et al. postulated altered liver mitochondrial DNA (mtDNA) content is an early event preceding mitochondrial dysfunction and irreversible liver damage in NAFLD [39]. In their study, they fed mice with a high fat or a Western diet for 16 weeks and determined the cellular copy number of mtDNA. The mtDNA content increased in the high fat group, while the mitochondrial oxidative phosphorylation system and mtDNA replication machinery were slowed down. This was accompanied with increased mtDNA-induced inflammatory pathways [39]. These data confirm the notion that diet-induced changes in liver mtDNA can occur in a relatively short time and are correlated to disease progression.

## 11. Targeted Therapies against Liver Fibrosis

Presently, a main research focus in fibrosis research is the development of targeted therapies with high liver specificity that are tolerated and promote the removal of excess interstitial matrix. In this Special issue, two reviews discuss the most recent advances in using viable cells, magnetic particles, or controlled magnetic forces in therapy of hepatic fibrosis [40,41]. In the first contributions, the authors explain why new discoveries on liver embryogenesis and adult liver regenerative mechanisms provide hints for new therapeutic targets and advanced treatments. In particular, pluripotent stem cells, diverse progenitor cells, and primary cells or established cells lines manipulated in vitro are supposed as effective means to generate cells for transplantation to promote liver regeneration and reversal of liver fibrosis [40]. Similarly, the second review highlights present experimental applications of magnetic nanoparticles in medicine and biotechnology with special emphasis on the selective targeting of activated HSC [41]. The available strategies have potential pitfalls, but also a host of opportunities for development of novel treatment option.

## 12. Animal Models in Hepatic Fibrosis Research

In biomedical research, there is an urgent need to improve animal models to better reproduce and mimic the disease patterns of human inflammatory disease [42]. This is also considered in this Special Issue containing three contributions introducing novel animal models to analyze special aspects of hepatic fibrogenesis. A new model of NASH in male rats uses a multi-hit protocol that combines the administration of a high fat and high-cholesterol diet with carbon tetrachloride inhalation and oral phenobarbital added to the drinking water [43]. To evaluate their model, the authors performed a large number of experiments; they show that rats treated with this regimen developed full characteristics of advanced human NASH after 10 weeks and NASH with cirrhosis after 24 weeks of treatment. In another article, Hempel and colleagues aimed to study the impact of bone marrow-derived fibrocytes for pathogenesis of hepatic fibrosis. Therefore, they developed a new model in which these cells were depleted utilizing a herpes simplex virus thymidine kinase/ganciclovir suicide gene strategy [44]. Mice depleted for these cell types showed significant lower fibrosis than control mice when subjected to thioacetamide treatment confirming fibrocytes as critical cells involved in the pathogenesis of hepatic fibrosis. Although the detailed mode of action was not unraveled, it is obvious that this model will be extremely helpful for further studies investigating aspects of fibrocyte biology. A third contribution by Guldiken et al., crossbred two established mouse lines that either were depleted for the Homeostatic Iron Regulator gene (*HFE*) or transgenic for the PiZ variant of α1-antitrypsin, which in humans predispose for pulmonary emphysema and liver disease. The authors could show that the loss of *HFE* had no impact on PiZ variant accumulation, iron accumulation, or development of hepatic fibrosis [45]. Although only tested in a small sample size in humans, the authors could further verify that *HFE* mutations are not associated with increased liver injury in patients carrying the homozygous PiZ variant.

## 13. Profibrogenic Cell Subpopulations in the Pathogenesis of Hepatic Fibrosis

Recent work from many laboratories has shown that HSC/MFB in healthy and diseased liver is not just a unique cell fraction, but rather, a heterogenous population made up of a number of different cell subsets [46]. The report of Krenkel et al. now characterized the heterogeneity of these liver mesenchymal cells by single cell RNA sequencing [47]. To do so, the authors isolated quiescent HSC and activated MFB from mice challenged with carbon tetrachloride for 3 weeks. The authors found that resting HSC formed a highly homogenous population, activated MFB converted into heterogenous subpopulations characterized by the differential expression of chemokines and collagens. These results support the notion of a yet unrecognized heterogeneity of MFB in vivo and suggest that the different subsets might have specialized functionality during liver fibrogenesis. The complexity of HSC is further highlighted in an article showing increased production of extracellular vesicles (EV) in cells that have made the phenotypic switch to MFB [48]. Comparative proteomic analysis of EV from activated primary mouse and human immortalized HSC cell line LX-2 showed similar EV protein signatures. Most interestingly, fibrogenic gene expression in fully activated HSC was suppressed when cells were incubated with EV isolated from relatively quiescent HSC, but not when subjected to EV isolated from passaged, fully activated HSC (i.e., MFB). These findings suggest a critical role of EVs in fine-tuning fibrogenic pathways in the liver.

During liver damage, various immune cells are triggered to produce high quantities of cytokines, chemokines, and enzymes that further provoke liver damage. Presently, mast cell involvement in the pathogenesis of hepatic fibrosis is becoming more accepted as reviewed in a contribution of this Special Issue [49]. They are present in healthy liver, and to a larger degree in diseased liver. Elevated numbers of hepatic mast cells were connected to the pathogenesis of many liver disorders. As outlined in this article, there are many therapeutic options for selective targeting of mast cells in liver fibrosis. However, the efficacy and benefits of the different compounds in therapy of hepatic fibrosis has not been systematically tested yet.

## 14. Communication among organs in Pathogenesis of Hepatic Fibrosis

Increasing evidence suggests alterations in mutual biological communication between distant organs as additional factors negatively impacting organ homeostasis, systemic inflammatory response, and fibrosis. The ‘heart-liver axis’ for example mediates the bidirectional communication between heart and liver and affects many aspects of inter-cellular and inter-organ communication related to inflammation and systemic energy metabolism. El Hadi and colleagues explain how acute and chronic heart failure can lead to acute hepatic injury and chronic congestive hepatopathy and vice versa, how liver disease can provoke the development of heart systolic and diastolic dysfunctions [50]. As the authors mentioned, early recognition of clinical signs and symptoms of simultaneous heart and liver injury has led to important benefits in terms of reduction of morbidity and mortality [50]. Similarly, Buniatian et al. points to a close connection of the brain in the pathogenesis of liver fibrosis [51]. The authors report that amyloid-β is not only crucial in forming amyloid plaques during Alzheimer’s disease, but has also antifibrotic activities during pathogenesis of liver fibrosis. Amyloid-β is efficiently internalized by HSC and provokes reduced expression of profibrogenic markers including α-smooth muscle actin, type I collagen, and TGF-β. The authors also provide evidence that amyloid-β upregulated sinusoidal permeability. These findings conclusively suggest that this short peptide exerts antifibrotic functions by both autocrine and paracrine effects on HSC and sinusoidal endothelial cells. Moreover, clinically more importantly, it is likely that efforts to lower its accumulation in the brain could have adverse effects on the liver [51]. The same group investigated aspects of α_2_-adrenergic receptor signaling [52]. This receptor system consists of three homologous subtypes (α_2A_, α_2B_, α_2C_) and has important roles in catecholamine signaling in different structures of the central nervous system. Interestingly, the different subtypes showed differential expression in carbon tetrachloride or bile duct-ligated mice, while the α_2_-adrenoblocker mesedin reduced profibrogenic markers and α_2A_ expression in culture murine HSC [52]. Moreover, mesedin enhanced expression of eNOS and suppressed TGF-β expression in a human hepatic sinusoidal endothelial cell suggesting α_2_-adrenergic receptor system to contribute to liver fibrosis by alleviating HSC activation and increasing the permeability of liver sinusoids during liver injury [52]. It is noteworthy that targeting the α_2_-adrenergic receptor system will have potential therapeutic implications.

The Special Issue includes novel findings of important anatomic aspects of liver fibrosis. Ghallab et al. used a toxic (carbon tetrachloride for 2, 6, and 12 months) a surgical (bile duct ligation for 21 days) and a genetic (*Mdr2*^−/−^) mouse model to investigate how liver fibrosis influences lobular zonation [53]. The authors carried out an extensive set of experiments and come to the conclusion that liver fibrosis of different etiologies leads to periportalization of liver lobules that occurs as a common response to percentral and also periportal damage [53]. Significantly, by use of functional genomics, the authors were able to compile a consensus list of periportal and pericentral genes that are relevant in mediating metabolic zonation.

## 15. Concluding Remarks

This Special issue received a high number of submissions showing that liver fibrosis is still a hot topic in Hepatology research. Therefore, *Cells* commissioned me to edit a second Special Issue on that discipline. The Special Issue “*Cellular and Molecular Mechanisms Underlying the Pathogenesis of Hepatic Fibrosis 2020*” is now open for submission. Research articles, reviews, or shorter perspective articles from experts on all aspects related to this topic are highly welcomed.
